# A Novel Method of Automatic Plant Species Identification Using Sparse Representation of Leaf Tooth Features

**DOI:** 10.1371/journal.pone.0139482

**Published:** 2015-10-06

**Authors:** Taisong Jin, Xueliang Hou, Pifan Li, Feifei Zhou

**Affiliations:** 1 School of Information Science and Engineering, Xiamen University, Xiamen, 361005, China; 2 School of Life Sciences, Xiamen University, Xiamen, 361005, China; The National Orchid Conservation Center of China; The Orchid Conservation & Research Center of Shenzhen, CHINA

## Abstract

Automatic species identification has many advantages over traditional species identification. Currently, most plant automatic identification methods focus on the features of leaf shape, venation and texture, which are promising for the identification of some plant species. However, leaf tooth, a feature commonly used in traditional species identification, is ignored. In this paper, a novel automatic species identification method using sparse representation of leaf tooth features is proposed. In this method, image corners are detected first, and the abnormal image corner is removed by the PauTa criteria. Next, the top and bottom leaf tooth edges are discriminated to effectively correspond to the extracted image corners; then, four leaf tooth features (Leaf-num, Leaf-rate, Leaf-sharpness and Leaf-obliqueness) are extracted and concatenated into a feature vector. Finally, a sparse representation-based classifier is used to identify a plant species sample. Tests on a real-world leaf image dataset show that our proposed method is feasible for species identification.

## Introduction

Because of a shortage of skilled subject matter experts, traditional species identification is tedious and difficult. The development of ubiquitous technologies, such as digital cameras and portable computers, has made automatic species identification possible. Specifically, for a large scale dataset of plant species, traditional species identification requires significant manual effort. It is unlikely that traditional species identification methods are practical; therefore, automatic species identification methods are more and more crucial. Leaf characters, involving shape, are used extensively in traditional text-based taxonomic keys for plant identification, which leads leaf shape to be the first and most commonly studied leaf feature.

### Leaf shape feature

Ingrouille’s work [1], for example, is one of the earliest efforts in automatic species identification, where twenty-seven leaf shape features were extracted to identify oak species. The following five shape features are also used for species identification: Fourier descriptors [2,3], contour signatures [4,5], landmark [6,7], quantitative shape descriptors [8,9] and fractal dimensions [10].

### Leaf venation feature

The second most commonly studied feature is leaf venation, also referred to as the leaf Vein. Leaf venation provides leaves with a specific structure, which may be useful to identify some species. The key is to effectively extract leaf venation; in this regard, several methods [11,12] have been proposed to effectively extract leaf venation. However, there have been fewer attempts to analyze or compare leaf venation; furthermore, most methods exploited synthetic or manually extracted leaf venation images.

### Leaf texture feature

The third most commonly studied feature is leaf texture; different leaf texture extraction methods are proposed in [13,14]. Although the existing leaf texture based methods have shown promising performance for some species, experiments using those methods involved very limited datasets. Thus, it is difficult to evaluate and is impossible to scale up a large scale dataset.

### Leaf margin feature

The fourth most commonly studied feature is leaf margin. Despite being a useful feature of leaves in traditional species identification, leaf margin has seen very little use in automatic species identification due to the difficulty in acquiring quantitative measurements automatically. Currently, only a few methods using leaf margin are proposed. Clark et al.[15], for example, used manually taken measurements, such as tooth length and width, to help automate species identification. Zheng et al.[16] extracted three morphological leaf tooth measurements, namely the number, sharpness and inclination, for plant identification. Zheng et al. [17]also defined a function to extract leaf lobe features. Cope’s study [18] gives a recent comprehensive review on automatic species identification.

All the above methods demonstrate that automatic species identification is suitable for some species. However, the key issue to solve the problem of robust automatic species identification is how to deal with the different deformation of leaf character and the large and small inter-class variations that are typical of botanical samples. Even if the study focuses on a single genus, it may contain many species, each of which encompasses extensive variation between constituent populations. Thus, the existing methods are insufficient to identify the complex species; additional features must be incorporated into the current automatic species identification method.

On the other hand, the classifier is very crucial for obtaining promising performance of species identification. Existing classifiers, such as *K* Nearest Neighbor (*K*-NN) [9], Random Forest [11], Support Vector Machine (SVM)[19] have been employed to identify plant species. More recently, the sparse representation based classifier has shown promising performance in face recognition [20], image analysis [21], and other applications [22,23]. However, to the best of our knowledge, the classifier based on sparse representation has not yet been applied to plant species identification.

Inspired by the recent progress of species identification and the sparse representation based classifier, we propose, in this article, a novel automatic plant species identification method. Unlike existing methods, our proposed method is based exclusively on leaf tooth; whereas, leaf shape, venation and texture are discarded. The contributions of this paper are as follows:

1. The morphological measurements of four leaf tooth features are proposed. Our proposed measurements effectively distinguish between the top and bottom edges of a leaf tooth. In addition, the noise effect is also removed using the PauTa criteria. Thus, our method is more suitable for real-world applications.
2. A sparse representation based classifier is applied to plant species identification. In our proposed method, an overall dictionary is constructed, and the species of a test sample is decided by the projection coefficients in the dictionary.
3. To demonstrate the feasibility of our proposed method, we conducted experiments on a real-world plant species dataset. In particular, we compared our proposed method with the *K* nearest neighbor (*K*-NN)-based and BP neural network-based methods.

## Materials and Methods

### Image pre-processing

A digital image of a plant leaf is usually obtained by a digital camera or a scanner. Compared with a scanner, digitalization using a digital camera is more suitable for image processing. Thus, in our experiments, we used a digital camera to digitalize plant leaf images. Because leaves are rarely perfectly flat and are affected by shadows and noise, we applied, to the leaf image, pre-processing carried out as follows: Firstly, the color image was converted into a grayscale image. Secondly, a binary image was obtained via adaptive image thresholding on the obtained grayscale image. A Roberts cross operator was further applied to the binary image to obtain an edge image. Thirdly, in order to remove many non-leaf margin edges retained in the edge image (specifically in the region of leaf venation) the dilation operator in morphology operations was used to fill in these holes. In our experiments, the dilation operator effectively removed most non-leaf-margin edges. Finally, the thin operator in mathematical morphology operations was used to make the leaf edge as thin as one pixel.

**Fig 1:**
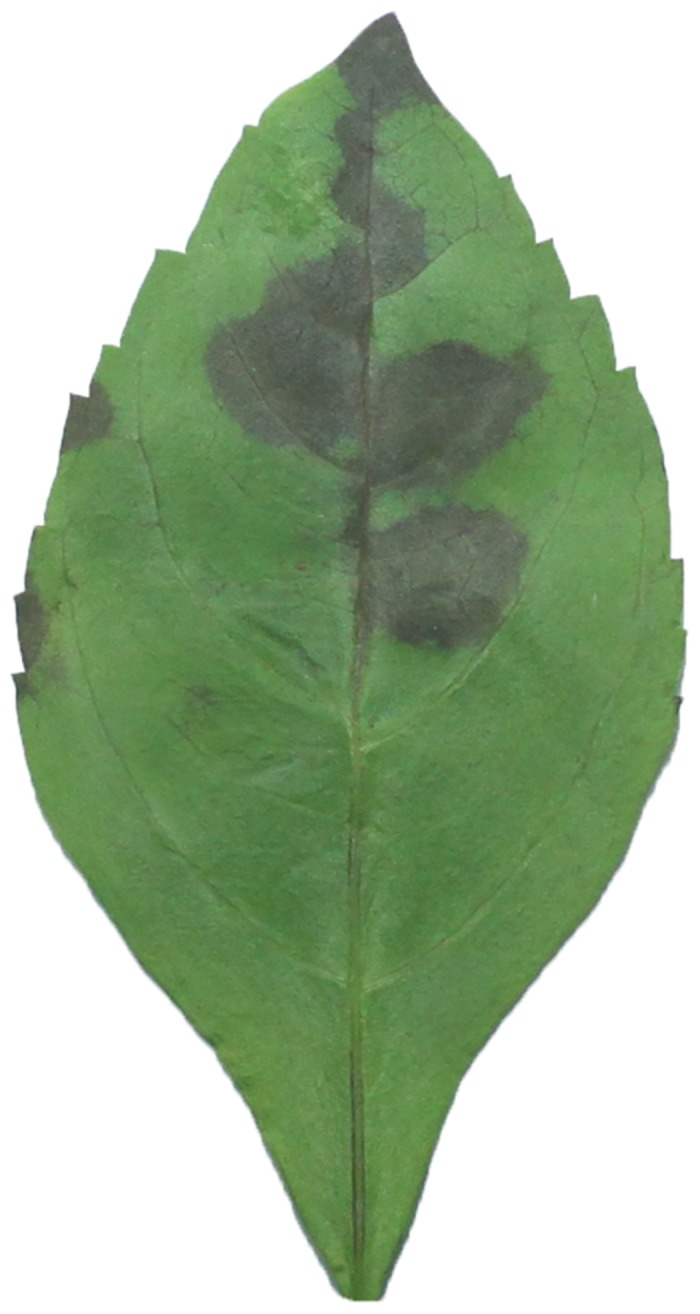
An example image for *Duranta repens* Linn.

**Fig 2:**
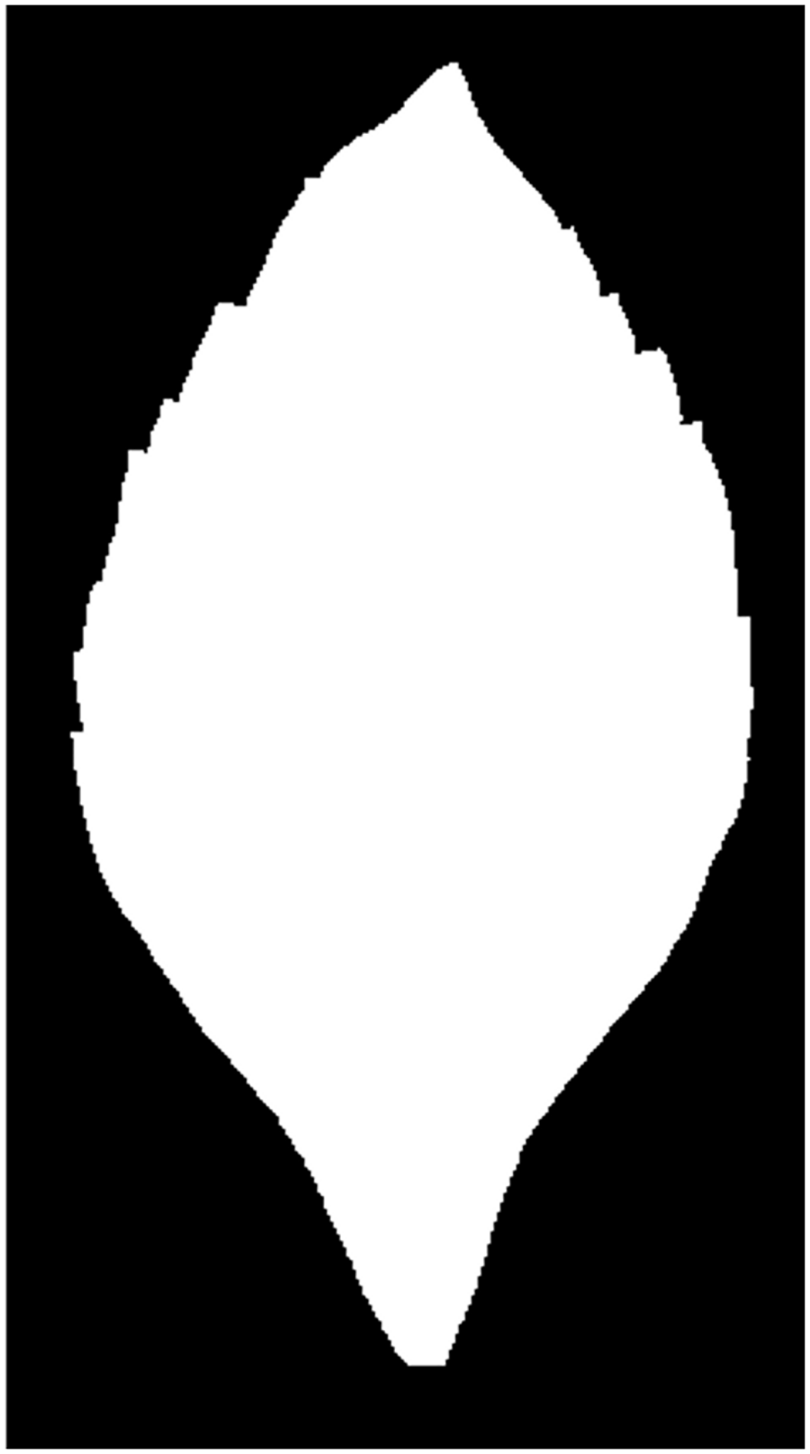
The obtained binary image.

**Fig 3:**
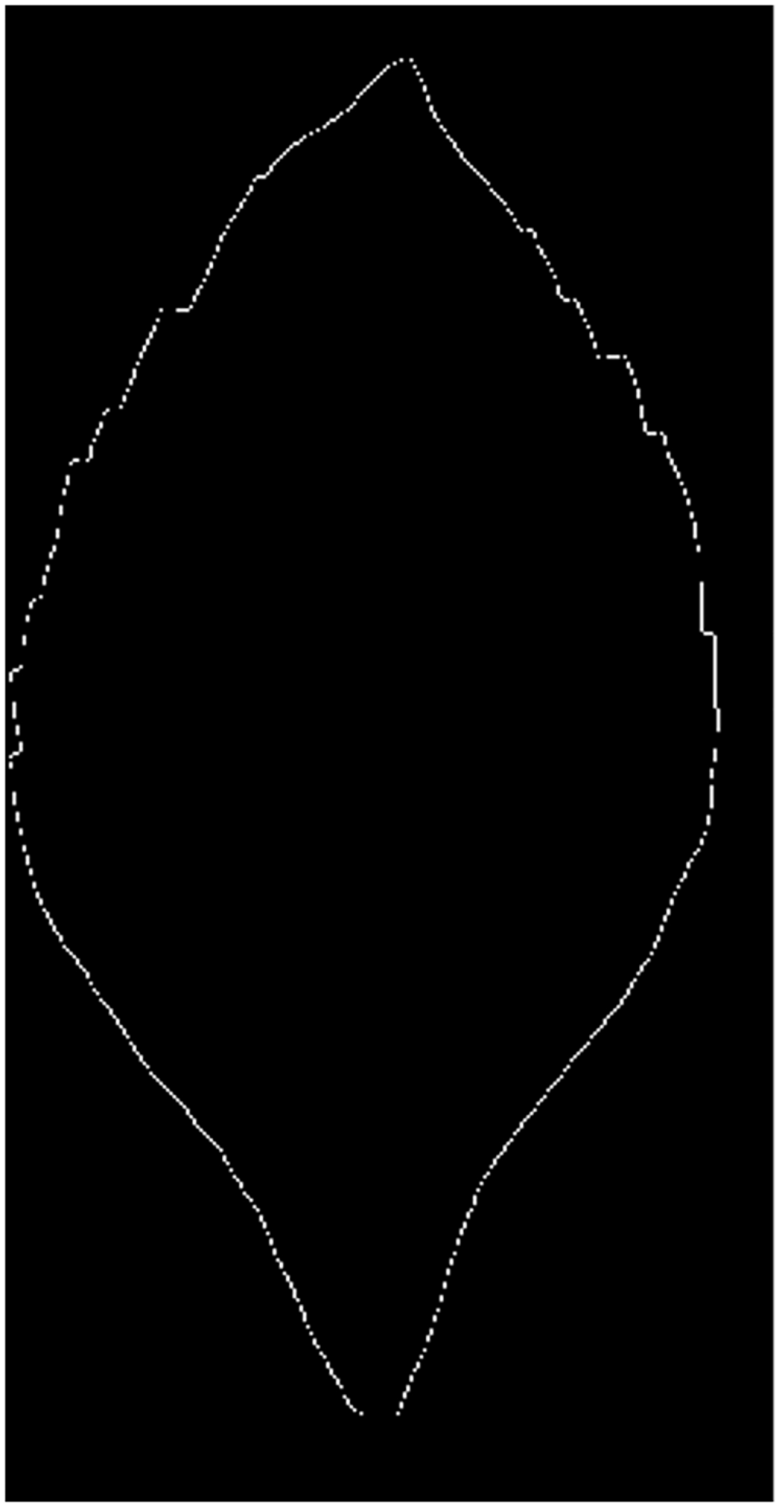
The obtained edge image via edge operator and thin operation.

All the aforementioned operations were implemented using the Matlab Toolbox. As shown in Figs 1, 2, 3, our proposed image pre-processing operations effectively extracted leaf margins.

### Extraction of image corners

Noise and illumination do not easily obscure image corners. Thus, image corners are commonly used to correspond to leaf teeth in the image. In our method, we also apply image corners to correspond to leaf teeth. For the different image corner extraction methods, researchers [16,24] have shown that Susan corner detection is more resistant to the effect of noise and is usually superior to other methods. Thus, we applied Susan corner detection to extract image corners from edge images. After extracting the image corners, most image corners corresponding to leaf teeth were detected. Due to the effect of noise, a few abnormal image corners may not correspond to leaf teeth. Thus, these abnormal image corners must be removed.

### The removal of abnormal image corners

We exploited the PauTa criteria to remove non-leaf-tooth image corners. For convenience, important notations are listed in Table 1. For each image corner, *C*_*i*_, we calculated two formulas according to the following PauTa criteria:||*C*_*i*_−*μ*_1_||>3*σ*_1_ and ||*C*_*i*_−*μ*_2_||>3*σ*_2_, where *μ*_1_ and *μ*_2_ are the means of horizontal coordinates and vertical coordinates of the image corners, respectively.

**Table 1:** List of important notations.

Notation and Description	
*μ*_1_	The mean of horizontal coordinates of the image corners
*σ*_1_	The standard deviation of horizontal coordinates of the image corners
*μ*_2_	The mean of vertical coordinates of the image corners
*σ*_2_	The standard deviation of vertical coordinates of the image corners
*μ*_3_	The mean of the third measures of all the leaf teeth
*σ*_3_	The standard deviation for the third measures of all the leaf teeth
*μ*_4_	The mean of the fourth measures of all the leaf teeth
*σ*_4_	The standard deviation for the third measures of all the leaf teeth
*T*a_i_	The third measure of the i-th leaf tooth
*Te*_i_	The fourth measure of the i-th leaf tooth
**D**_*i*_	The i-th species-specific sub-dictionary
**D**	The overall dictionary
**Y**	The sample to be identified
**X**_*i*_	The projection coefficient on the i-th species-specific sub-dictionary
**X**	The projection coefficient on the overall dictionary

To avoid one, or several, measurements of larger variation, abnormal image corners were removed based on the aforementioned Pauta criterion, i.e, if ||*C*_*i*_−*μ*_1_||>3*σ*_1_ or ||*C*_*i*_−*μ*_2_||>3*σ*_2_, then *C*_*i*_ was considered an abnormal image corner and was removed; otherwise, if ||*C*_*i*_−*μ*_1_||<3*σ*_1_ and ||*C*_*i*_−*μ*_2_||<3*σ*_2_, then *C*_*i*_ was considered a normal image corner and was preserved. Generally, zero to two abnormal image corners were removed in the implementation. Note that the operators ‘and’ and ‘or’ in the formulas are logical operators.

### Distinguishing the top and bottom edges of leaf teeth

**Fig 4:**
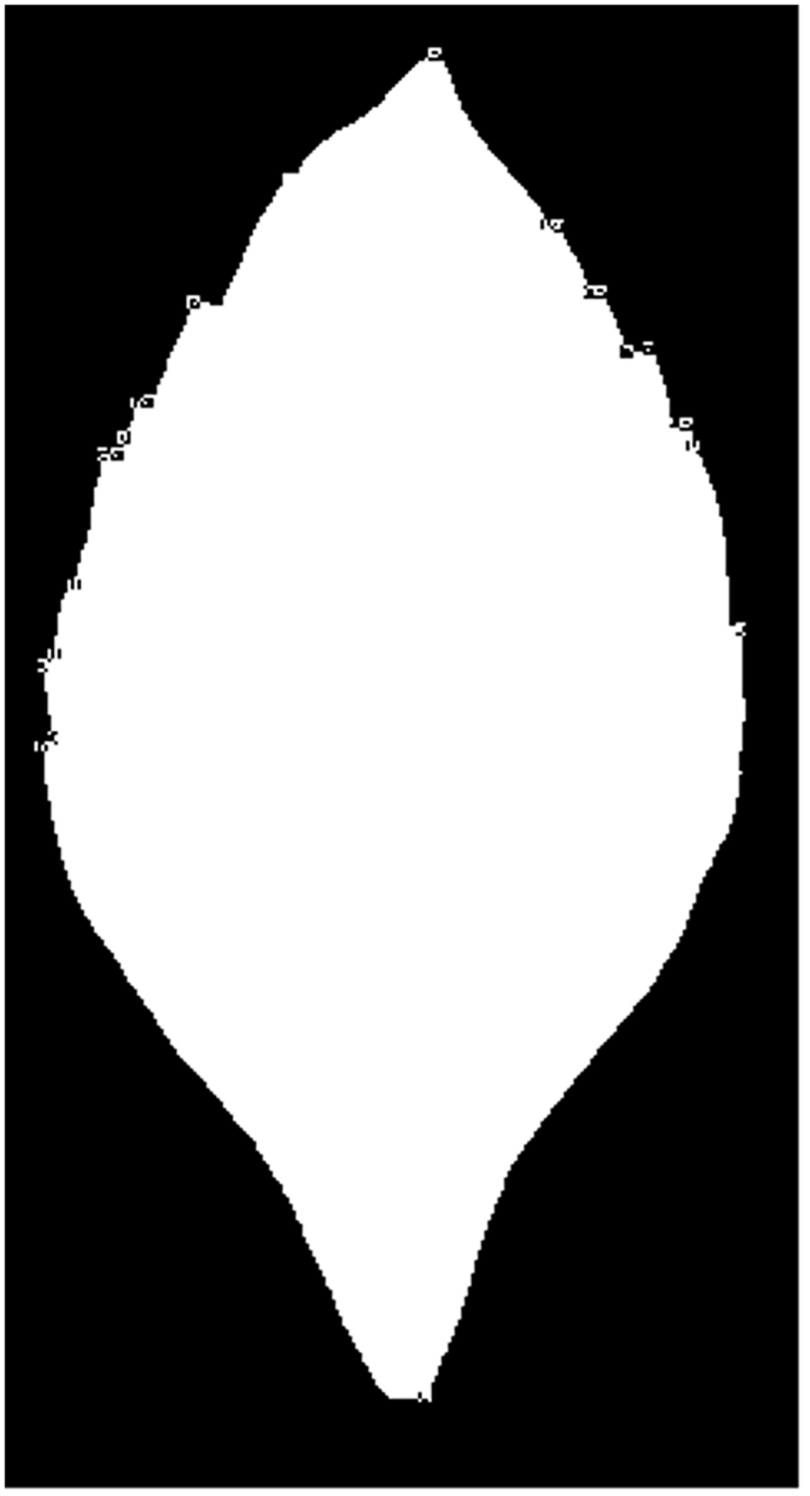
The image corners detected by the Susan method for Duranta repens Linn.

**Fig 5:**
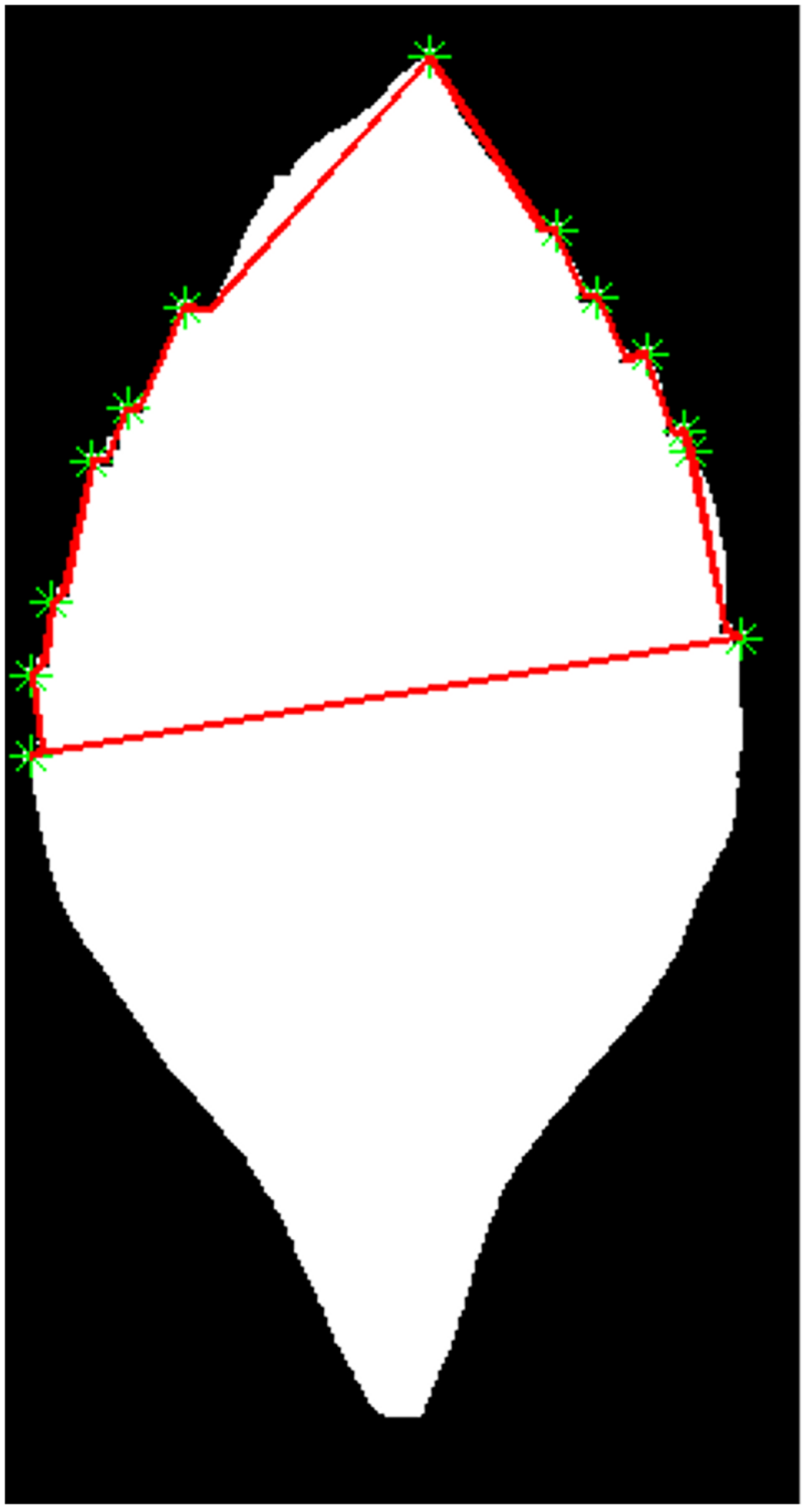
The image corners responding to leaf teeth.

After detection, image corners further corresponded to the bottom or top edges of leaf teeth. Firstly, a simple polygon was obtained by connecting the image corners with straight lines. Secondly, these image corners were distinguished in terms of the inner angle of a simple polygon, i.e., if the inner angle of the polygon is smaller than 180°, then the image corner vertex corresponds to the top edge of the leaf tooth; otherwise, it corresponds to the bottom edge. As shown in Figs 4 and 5, our proposed method correctly extracts the leaf teeth and also discriminates the top and bottom edges of the leaf teeth.

## Measurements of Leaf Tooth Features

After the leaf teeth are detected, the morphological measurements of four leaf tooth features are presented. In this section, we adopt the following four measurements as leaf tooth features:

**The first feature**, Leaf-num, is denoted by the first measure according to the total number of leaf teeth described by [16];

**The second feature**, Leaf-rate, is denoted by the second measure according to the ratio between the number of leaf teeth and the length of the leaf margin expressed in pixels.

**The third feature**, Leaf-sharpness, is measured as follows: For each leaf tooth, an acute triangle is obtained by connecting the top edge and two bottom edges of the leaf tooth. Thus, for a leaf image, many triangles corresponding to leaf teeth are obtained. In our method, the acute angle for each leaf tooth is exploited as a measure for plant identification. In detail, the third measure of the i-th leaf tooth, namely, *Ta*_*i*_, is calculated by the following formula:

$$T{\text{a}}_{\text{i}}=\text{arccos}\langle a,b\rangle ,$$ (1)

where **a,b** are the vectors obtained by connecting the top edge and two bottom edges of the i-th leaf tooth, respectively. In order to resist the effect of noise, we firstly remove the abnormal measure of the leaf teeth in terms of PauTa criteria as follows: the mean,*μ*_3_, and standard deviation, *σ*_3_, of all the third measures of the leaf teeth are calculated; next, for the i-th acute angle, *Ta*_*i*_ (*i* ϵ {1,2,⋅⋅⋅,*m*}), if||*Ta*_*i*_−*μ*_3_||>3*σ*_3_, then *Ta*_*i*_ is considered to be an abnormal measure and is removed. Otherwise, *Ta*_*i*_ is preserved for further processing. All the preserved measures are used for re-calculating the new mean as the third leaf tooth feature.

**Fig 6:**
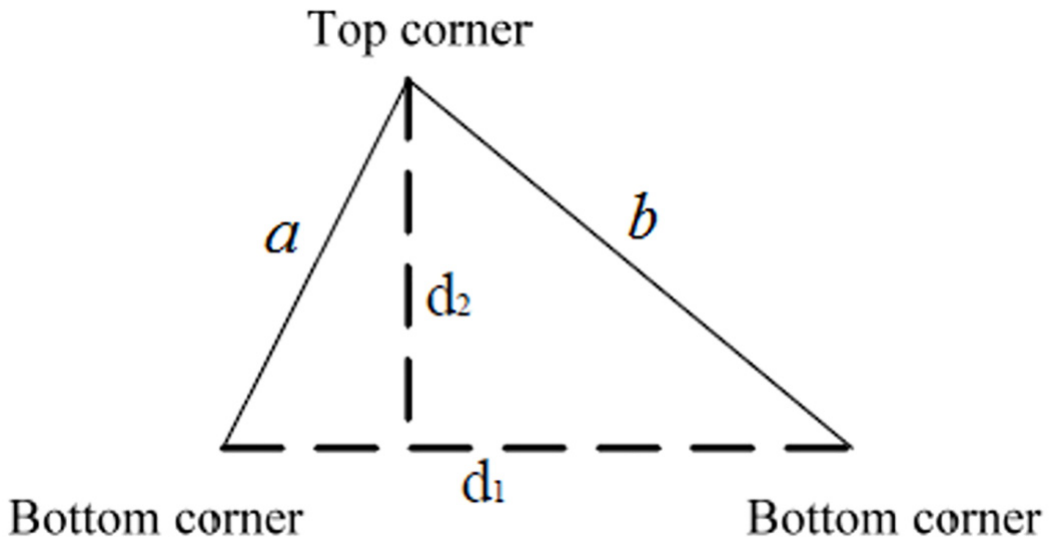
Illustration of the third and fourth leaf tooth features.

**The fourth feature**, Leaf-obliqueness, is measured as follows: Firstly, the fourth measure for the i-th leaf tooth,*Te*_*i*_, is formulated as

$$Te_{\text{i}}=\frac{\left|d_{1}\right|}{\left|d_{2}\right|}=\frac{|d_{1}{|}^{2}}{2\times S},$$ (2)

where |d_1_| and |d_2_| are the acute triangle’s base and height, respectively, obtained by the i-th leaf tooth; *S* is the area of the triangle. As such, the effect of noise leads to abnormal measures. Thus, by the PauTa criteria, the abnormal measures must be removed. The details of how to remove the abnormal measures are as follows: Firstly, the mean, *μ*_4_,and the standard deviation, *σ*_4_, of all the fourth measures of the leaf teeth are calculated; Secondly, for the i-th acute angle,*T*e_*i*_. (*i* ϵ {1,2,⋅⋅⋅,*m*}), if ||*T*e_*i*_−*μ*_4_||>3*σ*_4_, then *T*e_*i*_ is considered as an abnormal angle and removed; otherwise, *T*e_*i*_ is preserved for further processing. All the preserved measures are used to re- calculate the new mean as the fourth leaf tooth feature. The third and fourth features for one leaf tooth are illustrated in Fig 6.

## The Proposed Sparse Representation Based Classifier

After the four leaf tooth features are computed, they are concatenated into a feature vector. Then, a classifier is used to classify the feature vector as one plant species. Recently, a sparse representation-based classifier has shown promising performance for many applications. In our method, we incorporate a sparse representation-based classifier into plant species identification.

### Plant identification using the sparse representation based classifier

Sparse representation represents a signal as a linear combination of as few atoms as possible of a given dictionary. In other words, given the signals **y** ϵ ℝ^*n*^ and **D** ϵ ℝ^n×m^, the sparse representation problem is formulated as

$${\text{min}}_{x}{\left\|x\right\|}_{0,}\;s.\;t.\;y=Dx,$$ (3)

where ||**X**||_0_, is the *ℓ*_0_ norm of the coefficient vector, **x** ϵ ℝ^*m*^, i.e., the number of non-zero elements. The optimization of Eq (3) is a NP-hard problem, and direct optimization is not feasible. This problem is usually solved approximately by replacing *ℓ*_0_ norm with *ℓ*_1_ norm. Then, Eq (3) is transformed into the following optimization problem:

$${\text{min}}_{x}{\left\|x\right\|}_{1,}\;s.\;t.\;y=Dx.$$ (4)

The key issue of sparse representation is to obtain an over-completed dictionary that allows the sparse representation of the data samples with respect to the obtained dictionary. Many methods have been proposed to obtain such a dictionary. For instance, in [20], the entire training set is used as a dictionary, where excellent performance is shown for some real-world face image datasets. However, such a dictionary is not practical for large-scale image datasets. In [21], the visual feature vectors extracted from the images are used to build a dictionary, which significantly decreases the computational load. In the field of species identification, because of the large computational cost, it is unsuitable to use the whole training image set to obtain a dictionary. Thus, to build the dictionary, our proposed method applies the leaf tooth features extracted from the training set.

Suppose the training dataset consists of leaf images of q plant species, with *p*_*i*_ samples from the ith species; the total number of samples is $n=\sum_{i=1}^{q}p_{i}$. Firstly, the four leaf tooth features are calculated for each image. Furthermore, the four leaf tooth features are concatenated into a feature vector. Thus, a set of feature vectors is obtained for the entire training dataset. Let the feature vector extracted from the n-th image of the m-th plant species be **d**_mn_. Specifically, let all the feature vectors corresponding to m-th plant species be concatenated into a matrix $D_{\text{m}}=\left[d_{\text{m}1}d_{\text{m}2}\cdots d_{\text{m}p_{\text{i}}}\right]$, namely, the m-th species-specific sub-dictionary. Thus, the overall dictionaryD = [**D**_1_ **D**_2_⋅⋅⋅**D**_q_] is built by concatenating all the species-specific sub-dictionaries.

Given an overall dictionary, sparse representation means that any sample is represented linearly by a few atoms of the dictionary according to Eq (3), and a projection coefficient vector is obtained.

Ideally, for the projection coefficient vector **x** = [000⋯*x*_i1_ *x*_i2_⋯*x*_*im*_⋯000] of the sample on the dictionary, most of the coefficients are zero; only as few coefficients as possible, corresponding to its species-specific sub-dictionary, are not zero. In practice, because of the effect of noise, some coefficients, corresponding to other species-specific sub-dictionaries, are also non-zero. The re-constructed residuals on different species-specific sub-dictionaries are usually different. The smaller the residual on the species-specific sub-dictionary, the more likely it is that the sample belongs to the corresponding species. Thus, plant species can be identified according to the residuals on species-specific sub-dictionaries.

To remove the effect of noise, projection coefficients are computed on the dictionary according to Eq (4), i.e., species identification is transformed into the following optimization problem:

$$x^{*}=\text{arg}{\text{min}}_{x}{\left\|x\right\|}_{1}\;s.\;t.\;{\left\|y-Dx\right\|}_{}^{2}\leq \varepsilon ,$$ (5)

where *ε* is the threshold of the residual. This problem is transformed into the following problem:

$$x^{*}=\text{arg}{\text{min}}_{x}\;{\left\|y-Dx\right\|}_{}^{2}+\alpha {\left\|x\right\|}_{1},$$ (6)

where the parameter, *α*, indicates the trade-off between the sparsity of the solution and the reconstruction error. This is a constrained lasso problem; the detailed solution of Eq (6) is found in [23].

Given a test sample, **y** (to be identified), the method in [23] for solving the Lasso problem in Eq (6) is performed to obtain the projection coefficients. Additionally, the residuals on the species-specific sub-dictionaries are formulated as

$$r^{m}\left(y\right)={\left\|y-D_{m}x_{m}\right\|}_{}^{2},\;m=1,2\cdots \text{q,}$$ (7)

where *x*_*i*_ is the projection coefficient on the i-th species-specific sub-dictionary. Then, the species of the test sample, y, is identified as

$$Species\left(y\right)={\text{min}}_{m=1,2,\cdots \text{q}}r^{m}\left(y\right).$$ (8)

The proposed plant identification method has the following advantage: For adding a new plant species, our proposed method needs to only incorporate the plant samples of the corresponding species into an overall dictionary. Thus, it is easy to extend more plant species to realize the species identification task.

Our proposed method is described as follows:

Input: a normalized overall dictionary,**D**', and a test sample,**y**.
Output: the species of the test sample.
Step 1: Normalize the test sample.
Step 2: Calculate the projection coefficients of the test sample on an overall dictionary according to Eq (6).
Step 3: Calculate the re-constructed residuals on species-specific dictionaries according to Eq (7).
Step 4: Output the species of the test sample according to Eq (8).

### Implementation

To effectively implement our proposed automatic identification procedure, all the morphological measurements of the four leaf tooth features are first normalized to the range **[0,1]**. Then, to normalize an overall dictionary, the morphological measurements are scaled to be unitary. Furthermore, the corresponding features of the test image are also normalized to the range [0,1].

## Results, Discussion, and Conclusions

### The experimental setting

**Fig 7:**
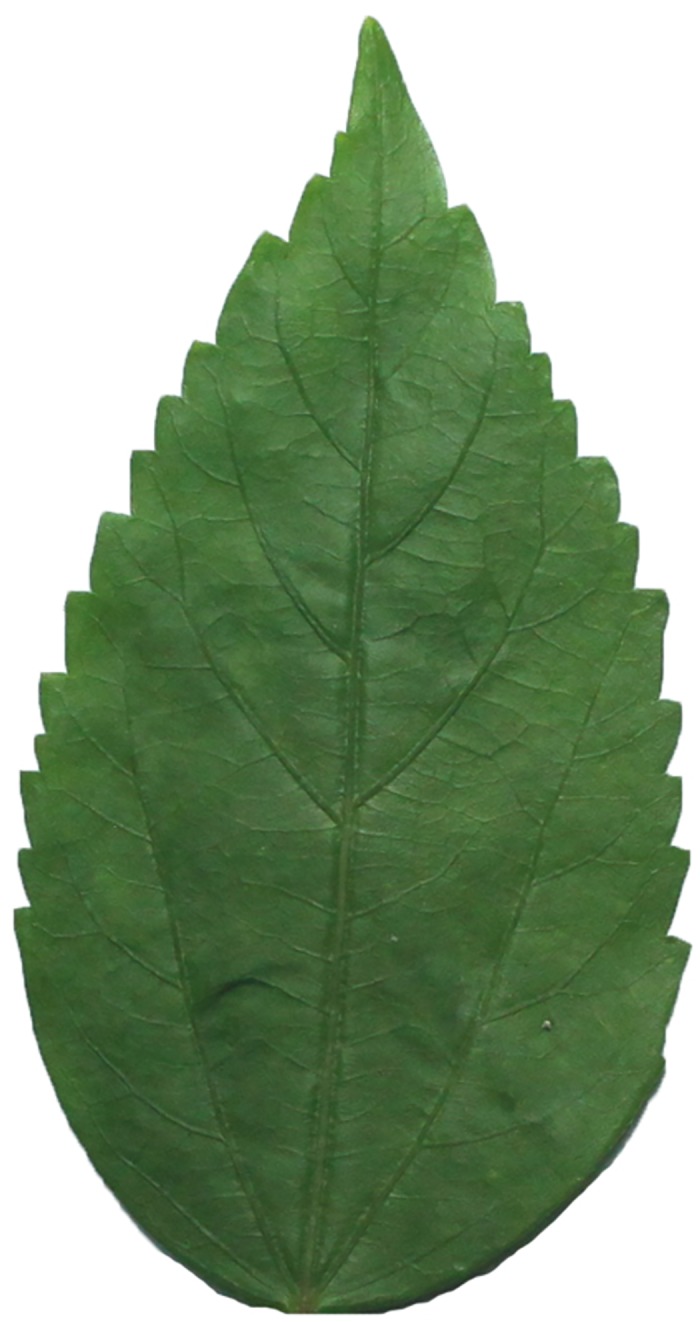
The example image for *Hibiscus rosa*-sinensis Linn.

**Fig 8:**
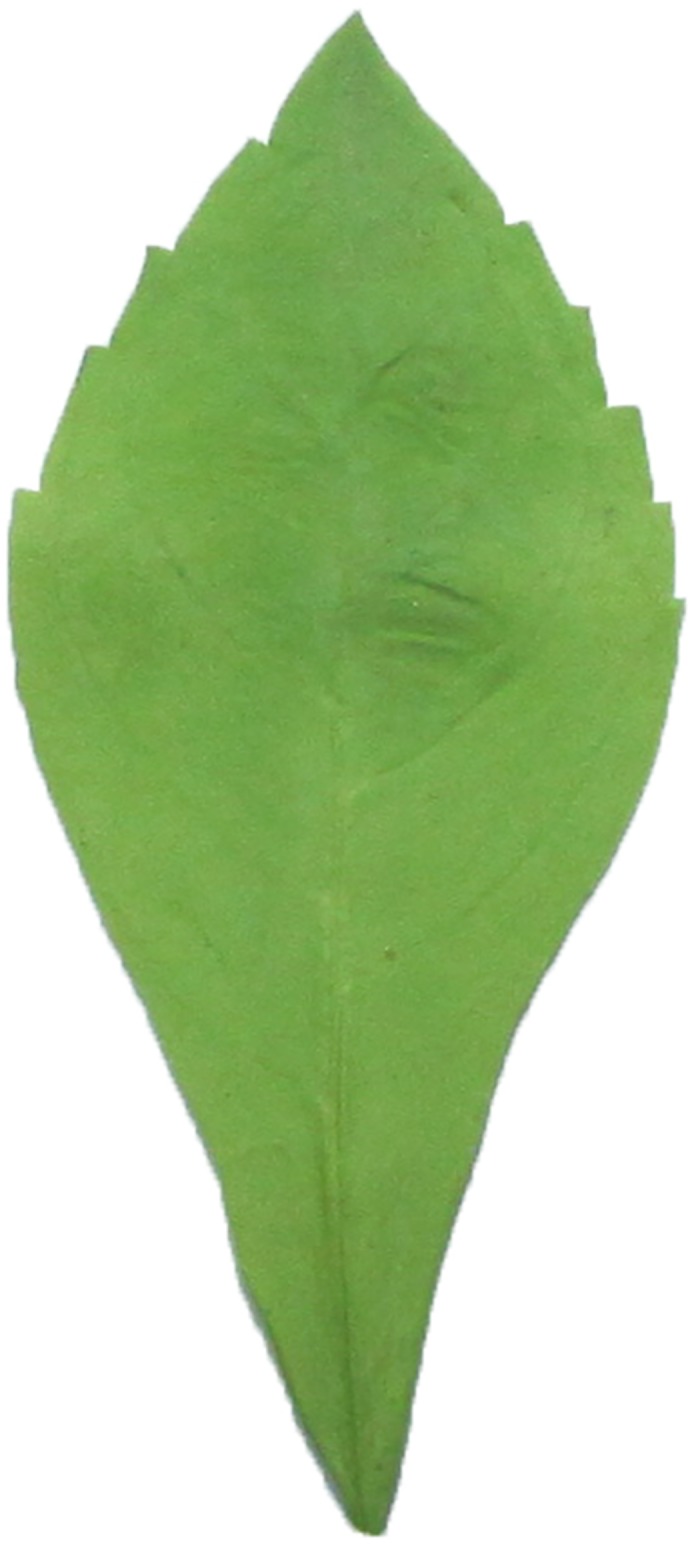
The example image for *Duranta repens* Linn.

**Fig 9:**
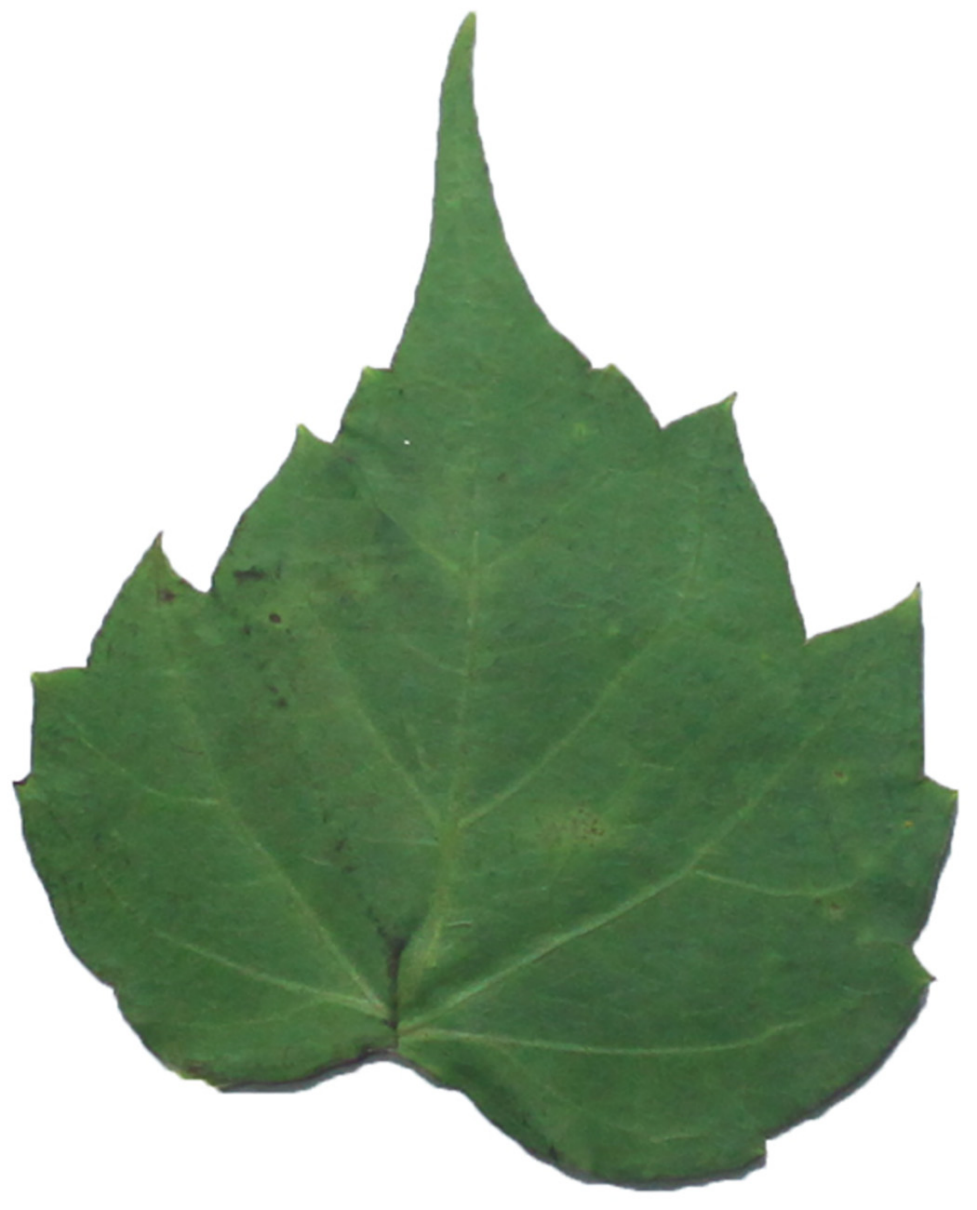
The example image for *Parthenocissus tricuspidata* (Sieb. et Zucc.) Planch.

**Fig 10:**
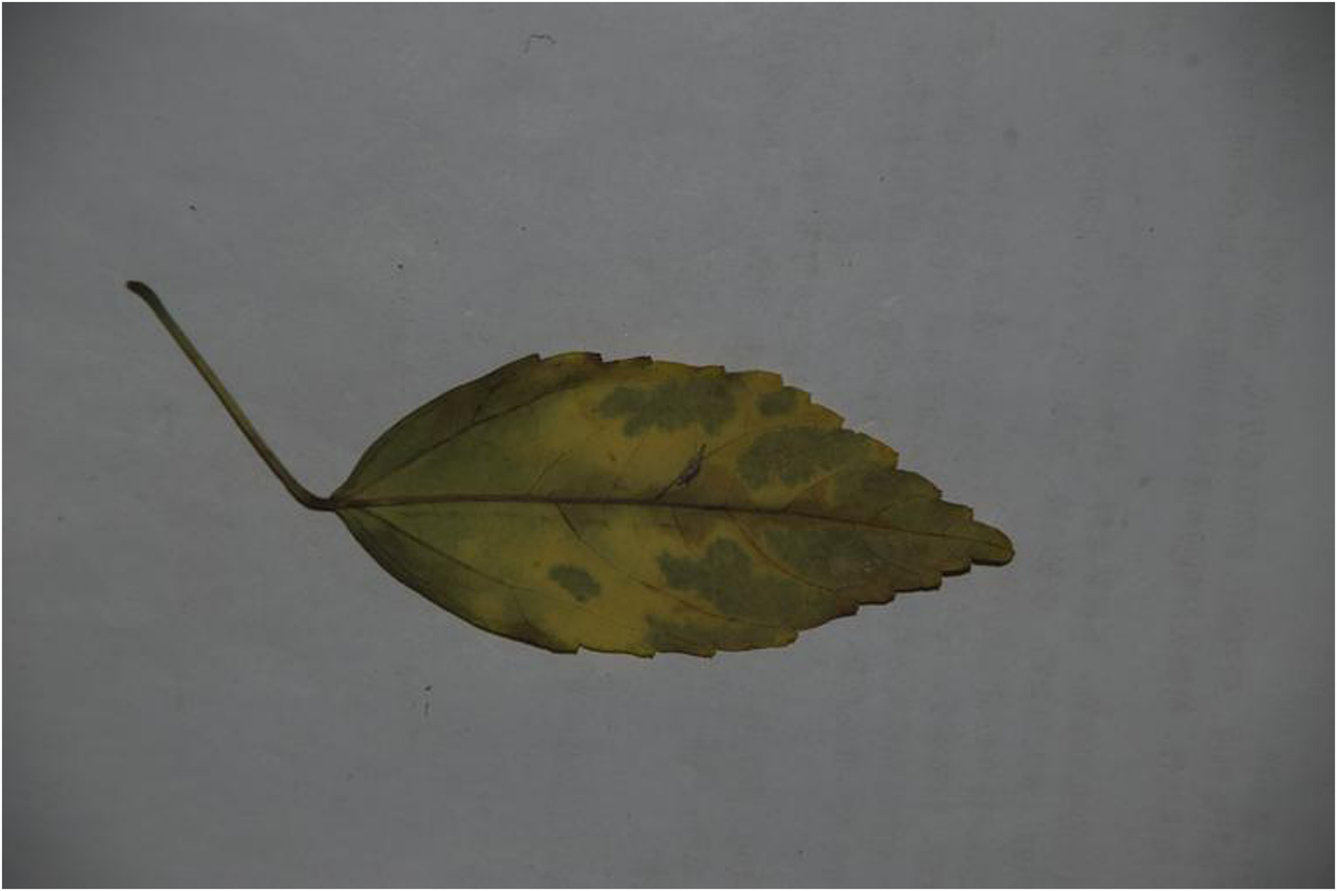
The example image for *Hibiscus schizopetalus* (Masters) Hook. f.

**Fig 11:**
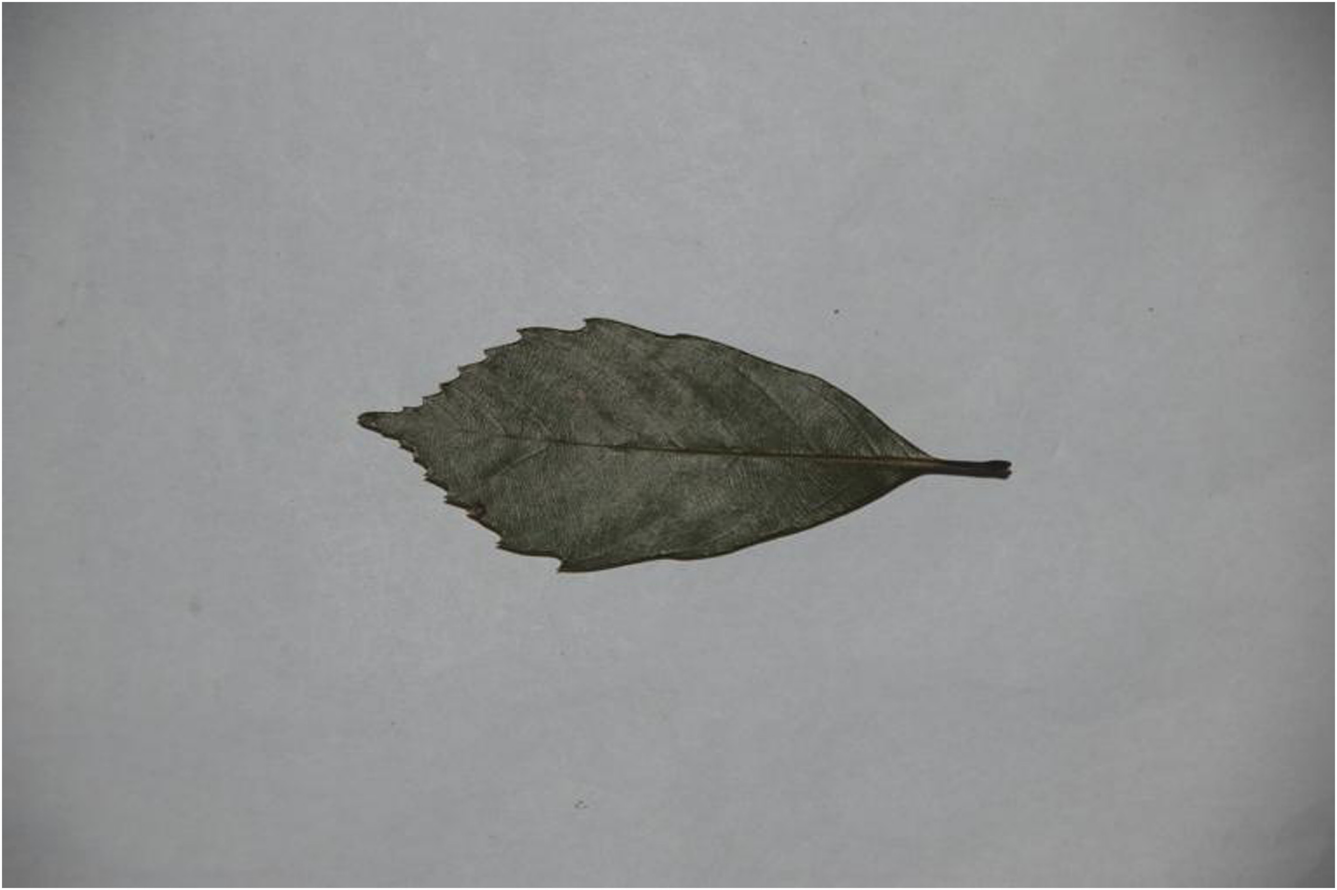
The example image for *Cyclobalanopsis glauca (Thunb*.*)* Oerst.

**Fig 12:**
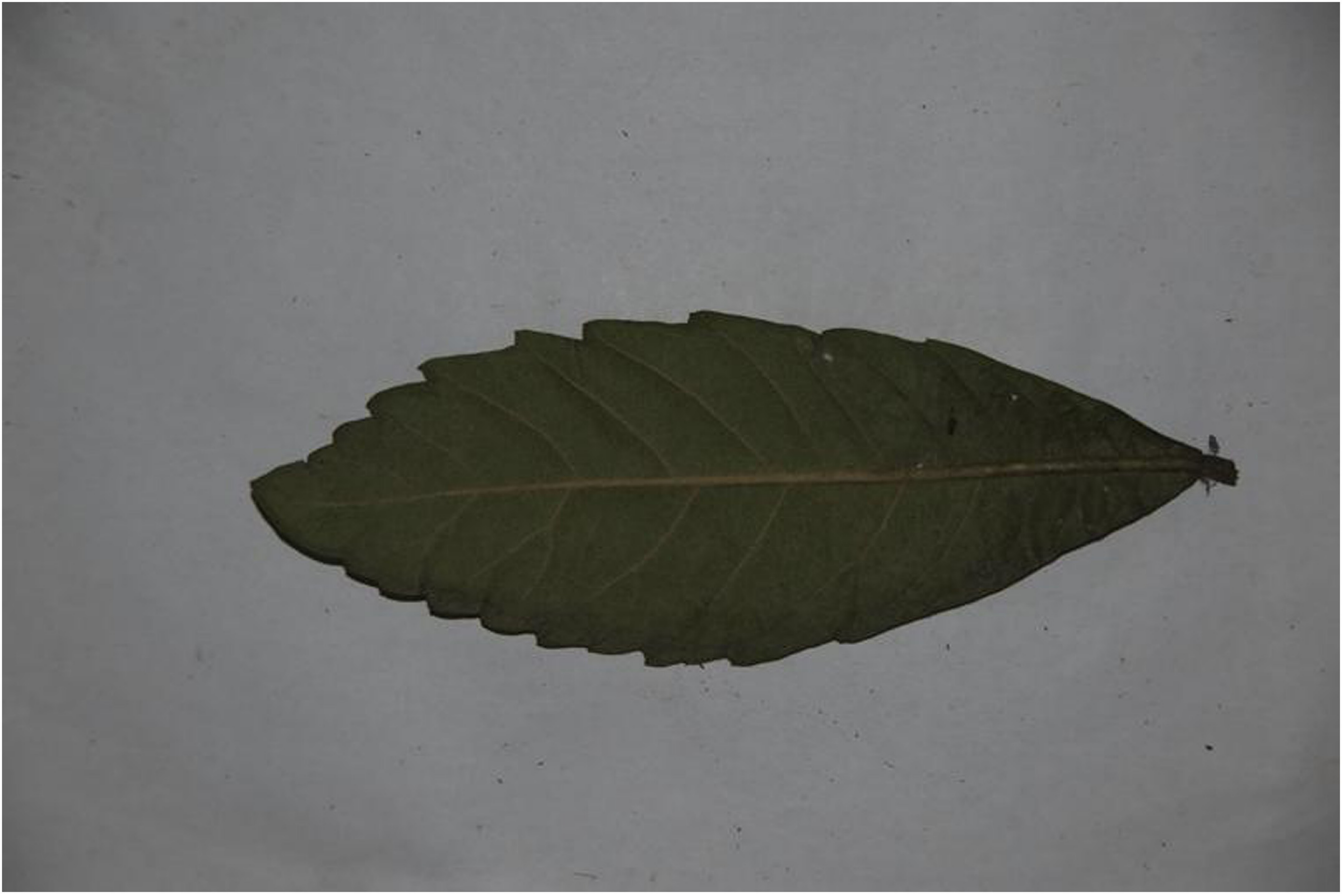
The example image for *Eriobotrya japonica* (Thunb.) Lindl.

**Fig 13:**
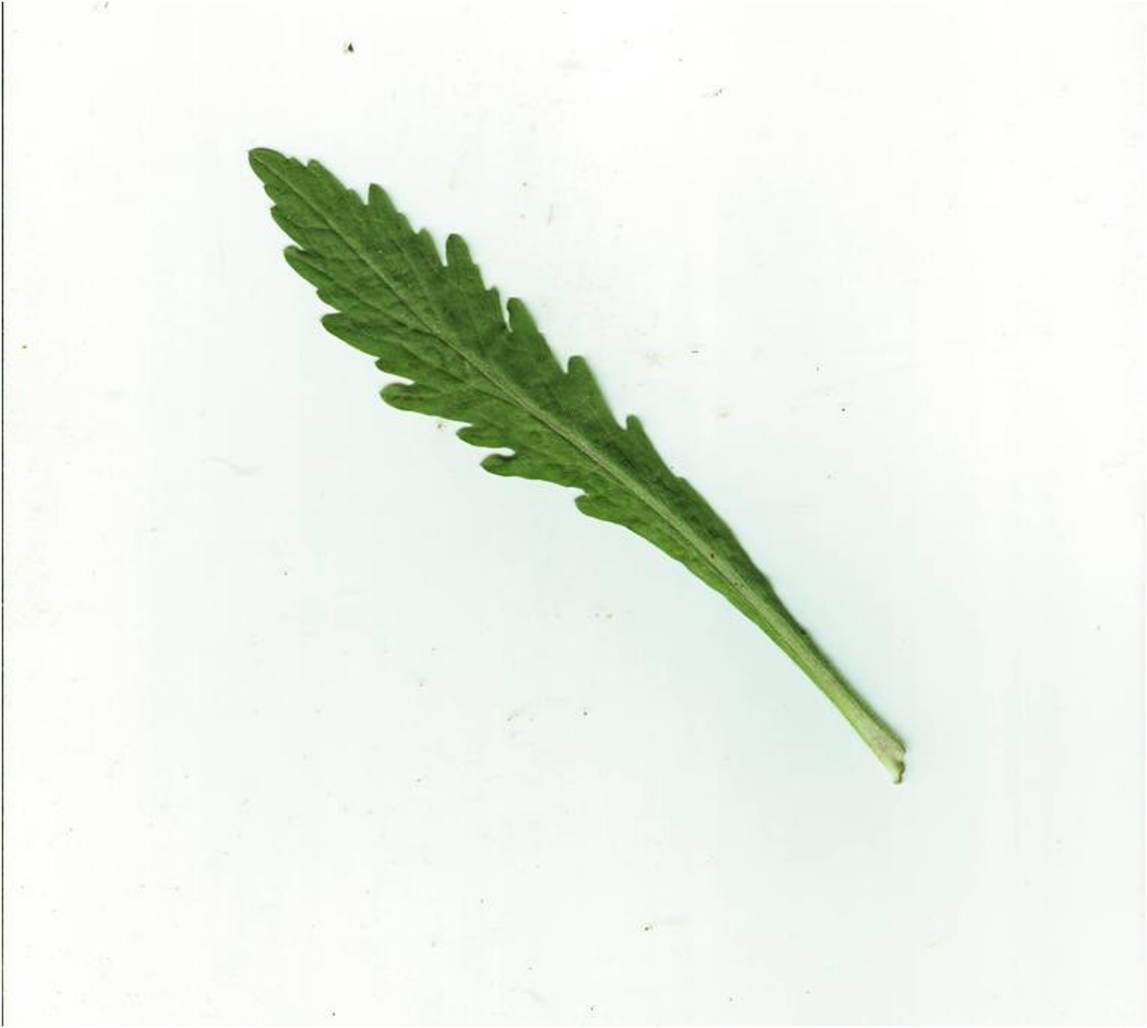
The example image for *Conyza canadensis* (L.) Cronq.

**Fig 14:**
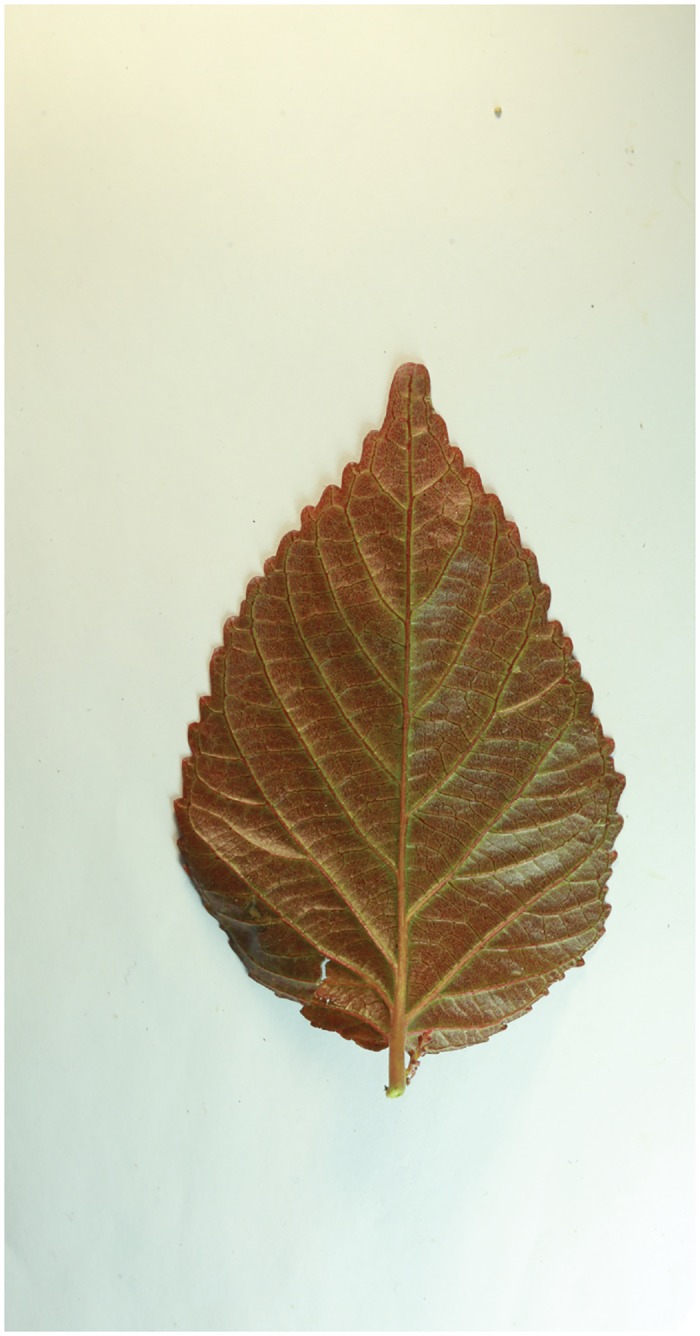
The example image for *Acalypha wilkesiana* Muell.-Arg.

Because different species own leaf characteristics with large and small inter-class variations, even if the study focuses on a single genus, it may contain many species, each of which encompasses much variation between constituent populations. Thus, we specifically select the species, which have similar leaf tooth character and observe the classification performance of the proposed method. To demonstrate the performance of our proposed method, we constructed, as an image dataset, a total of 700 leaf images (S1 File) from eight plant species. For each species, there are leaf images with variations in lighting, scale and background. The eight species have images as follows: (1) 54 images of *Hibiscus rosa*-sinensis Linn; (2) 96 images of *Duranta repens* Linn; (3) 54 images of *Parthenocissus tricuspidata* (Sieb. et Zucc.) Planch;(4) 124 images of *Hibiscus schizopetalus* (Masters) Hook. f; (5) 100 images of *Cyclobalanopsis glauca* (Thunb.) Oerst; (6) 82 images of *Eriobotrya japonica* (Thunb.) Lindl; (7) 124 images of *Conyza canadensis* (L.) Cronq; and (8) 66 images of *Amygdalus persica* Linn. Example images are shown in Figs 7, 8, 9, 10, 11, 12, 13, 14. A total of 350 images were used as the training dataset, where half of the images were randomly selected for each species. Then, the other 350 images were used as the test dataset.

### The plant identification test

Using our method, we first extracted the leaf tooth features from the training dataset and further built the overall dictionary; then, we ran our proposed method on the test dataset. The experimental results are listed in Table 2. The identification accuracy per species is reported over five independent runs, where the accuracy per species is the percentage of the successful identifications of each species relative to the number of leaves from the corresponding species.

**Table 2:** Accuracy of species identification of our proposed method.

Species	Mean accuracy ± Standard deviation (%)
*Hibiscus rosa*-sinensis Linn	75.0±3.4
*Duranta repens* Linn	79.3±2.1
*Parthenocissus tricuspidata* (Sieb. et Zucc.) Planch	76.3±3.2
*Hibiscus schizopetalus* (Masters) Hook. f.	76.6±2.9
*Cyclobalanopsis glauca* (Thunb.) Oerst	77.3±2.8
*Eriobotrya japonica* (Thunb.) Lindl.	75.5±4.5
*Conyza canadensis* (L.) Cronq.	74.7±1.7
*Acalypha wilkesiana* Muell.-Arg.	72.8±3.6

According to the experimental results (See Table 2), our proposed method achieved the following classification accuracies in specie identification: (1) Hibiscus rosa-sinensis Linn: 75.0%; (2) Duranta repens Linn: 79.3%; (3) Parthenocissus tricuspidata (Sieb. et Zucc.) Planch: 76.3%; (4) Hibiscus schizopetalus (Masters) Hook. f.: 76.6%; (5) Cyclobalanopsis glauca (Thunb.) Oerst.: 77.3%; (6) Eriobotrya japonica (Thunb.) Lindl.: 75.5%; (7) Conyza canadensis (L.) Cronq.: 74.7%; and (8) Acalypha wilkesiana Muell.-Arg.: 72.8%. The experimental results in Table 2 show that our proposed method achieves an average classification rate greater than 76% for all species.

The classification performance should be significantly greater. A possible explanation is that the similar characteristics of leaf teeth of different species, to some extent, affect the classification performance of our proposed method. However, our proposed method is based exclusively on leaf teeth; leaf shape, veining and texture were discarded. We consider that the identification results are acceptable for automatic species identification. Moreover, the classification results can be further enhanced by introducing other leaf features such as leaf shape and leaf venation. Because we focused on leaf tooth features in this paper, we didn’t conduct tests using other leaf features.

### The statistics of leaf tooth features

The four leaf tooth features extracted from the example images (See Fig 4) are shown in Table 3. From the data in Table 3, we conclude the following: the leaf tooth features obtained by our proposed method are intuitively consistent with human observations. Thus, because traditional plant identification commonly considers leaf tooth to be effective, the four leaf tooth features obtained by our proposed method are feasible. It is to be emphasized here that the results obtained by our proposed method are not consistent with the real results; however, the identification test we conducted demonstrated that our method is effective for plant identification.

**Table 3:** The four leaf tooth features for four example images.

Species	leaf-num	leaf-rate	leaf-sharpness	leaf-obliqueness
***Hibiscus rosa-sinensis* Linn**	26	0.0137	112.2600	0.2094
***Duranta repens* Linn**	11	0.0068	105.5330	0.1438
***Parthenocissus tricuspidata* (Sieb. et Zucc.) Planch**	12	0.0072	97.4062	0.2456
***Hibiscus schizopetalus* (Masters) Hook. f.**	14	0.0086	101.4717	0.1242
***Cyclobalanopsis glauca* (Thunb.) Oerst**	12	0.0152	140.1123	0.1169
***Eriobotrya japonica* (Thunb.) Lindl.**	15	0.0138	133.2329	0.1342
***Conyza canadensis* (L.) Cronq.**	23	0.0208	80.8092	0.1914
***Acalypha wilkesiana* Muell.-Arg.**	80	0.0149	119.3086	0.2651

### The comparison with the other methods

To further compare our proposed method with existing methods, we applied two existing classifiers to the same training and test datasets. The common classifiers are the *K*-NN and BP Neural Network used for plant identification applications. *K*-NN is a non-parametric classifier, and the test sample is identified by a majority vote of its neighbors, with the sample being assigned to the species that is most common among its *K* nearest neighbors. BP neural network is a multiple-layer feed-forward network trained according to the error back propagation algorithm. In our test, the *K* Nearest Neighbor parameter is set to 2, and the BP Neural Network consists of four inputs, one output, and one hidden layer composed of ten neuron units; transfer functions of each unit adopt a logarithmic sigmoid transfer function.

The classification accuracy per species for all three classifiers, where the accuracy per species is the percentage of the successful identifications of each species relative to the number of leaves from the corresponding species, are reported over five independent runs. The species identification results for the classifiers are listed in Table 4.

**Table 4:** Species identification results using the different classifiers.

Classifiers	Mean accuracy ± Standard deviation (%)
**BP neural network**	73.2±3.3
***K*-NN**	72.3±2.5
**Our proposed classifier**	76.3±3.4

From the experimental results shown in Table 4, we observe the following: our proposed method outperforms the *K*-NN and BP neural network methods. By comparison, the total accuracy of species identification of our proposed method is improved by about 4% and 3%, respectively. The possible reason is that the sparse representation based classifier is more robust to the effect of noise and other factors such as illumination and shadow. Thus, our proposed classifier is feasible for plant identification.

## Conclusion

In this paper, we proposed a novel automatic plant species identification method using sparse representation of leaf tooth features. Our proposed method introduced four leaf tooth features for plant species identification. Then, a sparse representation-based classifier was used to identify the plant species for a test sample. We conducted the experiments on a real-world dataset, showing that our proposed method was feasible for plant identification. In future work, we plan to study the more complex features of leaf teeth. In addition, other features such as shape, color and texture will be incorporated into the sparse representation based plant species classification framework. Thus, the classification performance can be significantly enhanced.

## Supporting Information

S1 File Plant Species dataset. (RAR)
